# Mid-Term Recovery of Right Ventricular Function and Improvement of Left Ventricular Function After Da Silva Cone Procedure for Ebstein Anomaly

**DOI:** 10.3390/jcdd12070276

**Published:** 2025-07-17

**Authors:** Krithika Sundaram, Veenah Stoll, Luciana Da Fonseca Da Silva, Adam Christopher, Arvind Hoskoppal, Jacqueline Kreutzer, David Liddle, Laura Olivieri, Jacqueline Weinberg, Craig P. Dobson, José P. Da Silva, Tarek Alsaied

**Affiliations:** 1 Department of Pediatrics, UPMC Children’s Hospital of Pittsburgh, University of Pittsburgh School of Medicine, Pittsburgh, PA 15224, USA; 2 Division of Pediatric Cardiothoracic Surgery, Department of Cardiothoracic Surgery, University of Pittsburgh School of Medicine, Pittsburgh, PA 15224, USA; stollvk@upmc.edu (V.S.); dafonsecadasilval@upmc.edu (L.D.F.D.S.);; 3 Heart Institute, UPMC Children’s Hospital of Pittsburgh, Pittsburgh, PA 15224, USA; adam.christopher2@chp.edu (A.C.); hoskoppalak@upmc.edu (A.H.); jacqueline.kreutzer@chp.edu (J.K.); liddledw@upmc.edu (D.L.); olivierilj@upmc.edu (L.O.); jacqueline.weinberg@chp.edu (J.W.);; 4 Division of Pediatric Cardiology, Department of Pediatrics, UPMC Children’s Hospital of Pittsburgh, University of Pittsburgh School of Medicine, Pittsburgh, PA 15224, USA

**Keywords:** Ebstein anomaly, cone procedure, right ventricular function, left ventricular function, echocardiographic measures

## Abstract

Background: The Da Silva Cone procedure for Ebstein anomaly has dramatically improved tricuspid valve competence and clinical outcomes. However, preoperative left ventricular (LV) dysfunction and immediate postoperative right ventricular (RV) systolic dysfunction are frequently observed. While excellent valve outcomes are well established, recovery of biventricular function following the Cone remains less defined. This study aimed to evaluate longitudinal changes in RV and LV function postoperatively and over a minimum of six months post-Cone operation. Methods: A single center retrospective review of 134 patients who underwent Cone repair for Ebstein’s anomaly from 2016 to 2024 was performed. Echocardiograms were analyzed at three time points: preoperative (Time 1), hospital discharge (Time 2), and ≥6 months postoperative (Time 3). RV parameters included fractional area change (FAC), tricuspid annular plane systolic excursion (TAPSE), and tricuspid S′. LV parameters included left ventricular ejection fraction (LVEF), end-diastolic volume indexed to body surface area (LVEDVi), left ventricular stroke volume (LVSVi), and mitral E/E′. Subgroup analyses examined outcomes by prior Glenn, Starnes procedure, and degree of RV dilation. Paired two sample *t*-tests were used to compare serial measures. Results: Median age at surgery was 7.8 years (IQR: 2.3–17.7). All patients had discharge echocardiograms; 70 had follow-up studies at ≥6 months. RV function declined postoperatively with reductions in FAC (35% to 21%), TAPSE (2.0 to 0.8 cm), and S′ (13 to 5 cm/s), all *p* < 0.001. By Time 3, these measures improved (FAC to 29%, TAPSE to 1.3 cm, S′ to 7 cm/s) but did not fully return to baseline. LVEDVi and LVSVi increased significantly by Time 3 (LVEDVi: 47 to 54 mL/m^2^; LVSVi: 30 to 34 mL/m^2^; *p* < 0.001), while LVEF remained unchanged. Patients with prior Glenn or Starnes had greater Time 1 LV volumes and lower RV function, but by Time 3, most differences resolved. Moderate–severe preoperative RV dilation was associated with worse RV function at Time 2 and normalized by Time 3. Conclusions: The Da Silva Cone procedure leads to early postoperative RV dysfunction with partial recovery over the mid-term follow-up. Concurrently, LV filling and stroke volume improve, reflecting favorable interventricular interaction. These findings support echocardiographic surveillance to guide functional recovery post-Cone and inform patient counseling.

## 1. Introduction

Ebstein anomaly is defined as rotational displacement of the tricuspid valve with adherence of the inferior and septal leaflets to the ventricular myocardium and concomitant atrialization of the right ventricular (RV) wall. Without surgical repair, a proportion of these patients will progress to life-threatening RV failure and arrhythmias. The preferred surgical method at the University of Pittsburgh Medical Center (UPMC) for Ebstein anomaly is the Da Silva Cone operation. The operation includes circumferential leaflet delamination, rotation, and reattachment to the true tricuspid valve annulus [1,2].

The Cone procedure has been shown to improve outcomes in Ebstein anomaly and results in decreased tricuspid regurgitation (TR) with improved cardiac output, thus allowing forward flow through the right ventricle [1]. Prior to Cone, the RV is exposed to a high-volume load secondary to TR [3]. A subset of patients require volume unloading of the RV with the modified Starnes procedure, which also includes augmentation of pulmonary blood flow using a systemic to pulmonary shunt or bidirectional Glenn anastomosis [4]. Specifically, severe presentation in neonates is often palliated with a modified Starnes procedure to improve left ventricular (LV) hemodynamics and achieve future RV rehabilitation [5]. This no longer commits patients to a single ventricle pathway and subsequent Cone can successfully be performed in early childhood [6].

After the Cone, the RV has substantially decreased end-diastolic volume (EDV) due to the reduction in the TR and RV plication performed during the Cone procedure, and RV systolic dysfunction is frequently observed [7]. This dysfunction is thought to be multifactorial. The RV in Ebstein patients can have abnormal histologic structure, and the atrialized portion is commonly thin and poorly contractile. In addition, right bundle branch block and myocardial injury after valve delamination play a role in postoperative RV dysfunction [8]. Our study goal was to assess biventricular function in patients pre- and post-Cone procedure. We also aimed to evaluate the effect of RV dilation and prior Glenn or Starnes procedures on ventricular function.

## 2. Methods

### 2.1. Study Population

A single center retrospective chart review was performed on 134 consecutive patients who underwent Cone operation in our institution from 2016 to 2024. The study was approved by the University of Pittsburgh Institutional Review Board.

### 2.2. Transthoracic Echocardiograms

Transthoracic echocardiograms were performed per institutional protocol at 3 different time points for each patient. These were: preoperative echocardiogram within one month of the Cone operation in a majority of patients (Time 1), postoperatively at hospital discharge (Time 2), and at least six months postoperatively (Time 3). The degrees of TR and RV dilation were noted at Times 1 and 3. Right and left ventricular function parameters were obtained on the echocardiograms at Times 1, 2, and 3. As our institution is an international referral location for Ebstein surgery, many patients returned to their home institutions and thus Time 3 data was not available. For this, the electronic medical record systems were used to compile outside hospital echocardiograms. The parameters were also stratified at each time point by previous Glenn or Starnes procedure as well as the degree of RV dilation. LV size and function measures obtained included left ventricular ejection fraction (LVEF), body surface area (BSA) indexed end-diastolic volume (LVEDVi), mitral valve E/E′, and BSA indexed LV stroke volume (LVSVi). RV function measures obtained included fractional area change (FAC), tricuspid annular plane systolic excursion (TAPSE), and tricuspid S′. The parameters were compared across the three time points.

### 2.3. Clinical Data

Study data were collected and managed using REDCap electronic data capture tools version 15.1.1 hosted at the University of Pittsburgh [9,10]. REDCap (Research Electronic Data Capture) is a secure, web-based software platform designed to support data capture for research studies. Demographic data included mean age at surgery, sex, and previous surgeries performed. The degree of RV dilation was measured at Time 1 and Time 3. RV and LV parameters were obtained and compared across the echocardiograms at Time 1, Time 2, and Time 3. Data was analyzed using two sample paired *t*-tests comparing each of the three measures (Times 1 vs. 2, Times 2 vs. 3, and Times 1 vs. 3). A subgroup analysis was stratified by previous Starnes or Glenn procedures as well as degree of RV dilation. These data were analyzed with an independent sample *t*-test. A *p*-value less than 0.05 was considered statistically significant.

## 3. Results

### 3.1. Demographics

The study included 134 patients (52% female) who underwent the Cone operation at our institution between 2016 and 2024 (Table 1). The median age at surgery was 7.8 years (interquartile range (IQR): 2.3–17.7 years). The youngest age at surgery was 50 days, and the oldest was 61 years. Thirty-nine patients (29%) had previous cardiac surgery, including Glenn operation (n = 25, 19%), Starnes patch placement (n = 14, 10%), aortopulmonary shunt (n = 30, 22%), previous Cone at outside institution (n = 6, 4%), atrial septal defect (ASD) surgical closure (n = 8, 6%), and other tricuspid and pulmonary valve replacement or repair (n = 13, 8%) (Table 2). These procedures are not mutually exclusive. Additionally, 14 patients underwent catheterization-based intervention prior to the Cone operation at our institution, including patent ductus arteriosus stent (n = 6, 4%) and ASD device closure (n = 8, 6%). All patients had a follow-up echocardiogram before hospital discharge. A follow-up echocardiogram for Time 3 was available for review in 70 (52%) patients, as many had returned to their home institutions.

### 3.2. Operative Results

The median hospital length of stay was 6 days (IQR: 4, 8). Delayed chest closure occurred in 12 patients, 6 of whom required a brief period of extracorporeal membrane oxygenation support postoperatively. These were most commonly utilized to support the RV during initial recovery. There was zero 30-day mortality in the entire cohort with 3 patients suffering late mortality that was unrelated to the Cone operation.

### 3.3. Change in RV Function

**Figure 1:**
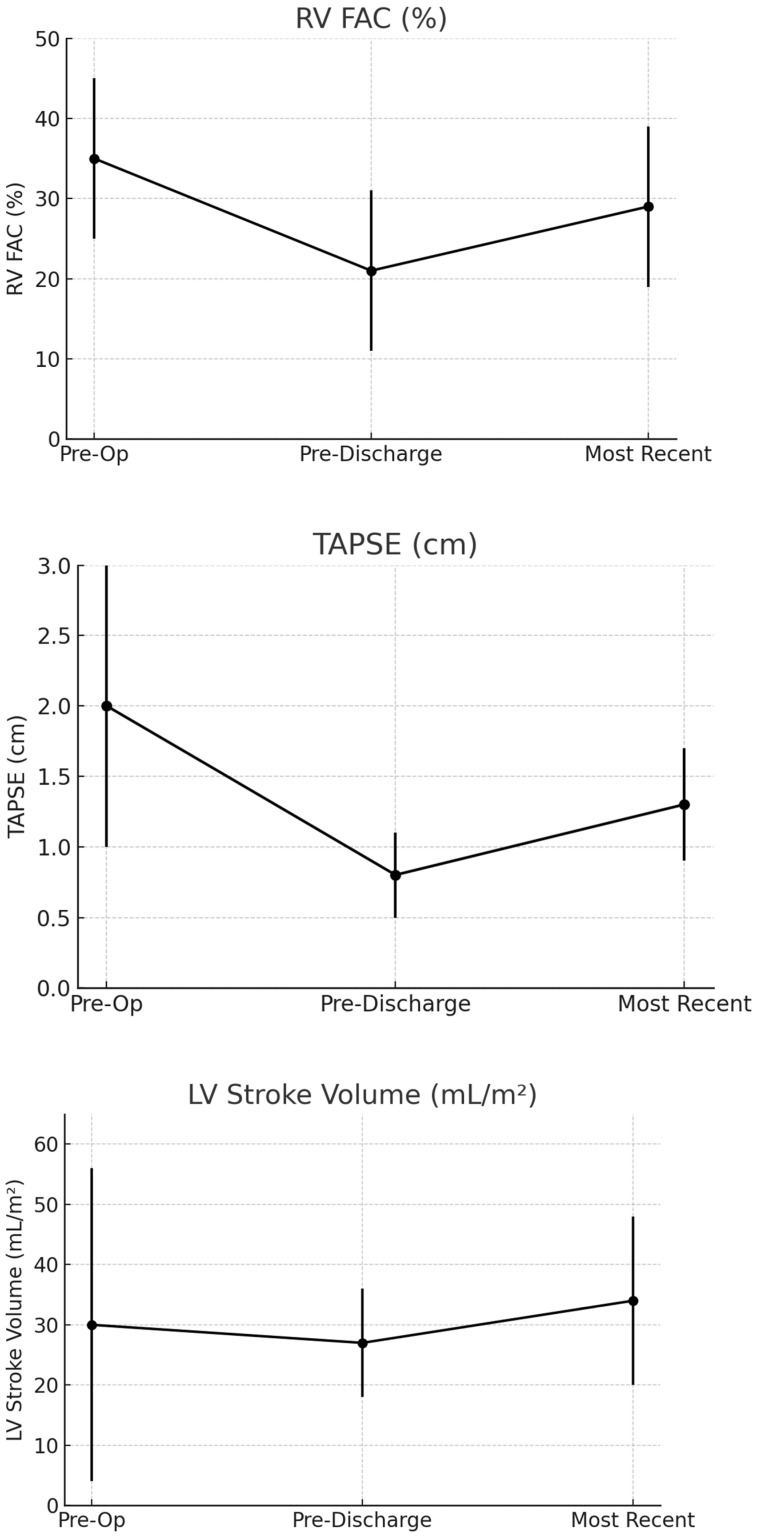
(**Top**) panel shows the RV FAC at the three different time points, with worsening at Time 2 and improvement at Time 3 (*p* < 0.04). (**Middle**) panel shows the TAPSE at the three different time points with worsening at Time 2 and improvement at Time 3 (*p* < 0.001). (**Bottom**) panel shows the LVSVi at the three different time points. There was a significant increase from Time 1 to Time 3 (*p* < 0.005) and from Time 2 to Time 3 (*p* < 0.03).

RV dilation was common prior to surgery: 23% had severe RV dilation, 26% had moderate RV dilation, 10% had mild RV dilation, and 2% had a mildly hypoplastic RV in the setting of a previous Starnes patch placement. Comparing the 134 preoperative echocardiograms (Time 1) versus discharge study (Time 2), there was significant decrease in TAPSE, tricuspid S′, and RV FAC immediately post-Cone with improvement of all measures on Time 3 (*p* < 0.001). These measures included FAC (Time 1: 35 ± 10%; Time 2: 21 ± 10%; Time 3: 29 ± 10%), tricuspid S′ (Time 1: 13 ± 6 (cm/s); Time 2: 5 ± 4 (cm/s); Time 3: 7 ± 2 (cm/s)), and TAPSE (Time 1: 2.0 ±1.0; Time 2: 0.8 ± 0.3; Time 3: 1.3 ± 0.4 cm) (Table 3). Figure 1 shows the changes in the TAPSE and RV FAC at all three time points.

### 3.4. Change in Left Ventricular Function

The LVEF from Time 1 to Time 2 had a statistically significant small increase (*p* < 0.01); however, there was no statistically significant change from Time 1 to Time 3 or Time 2 to Time 3. The LVEF measures were as follows: Time 1: 59% ± 6; Time 2: 61% ± 6; and Time 3: 61% ± 3. The LVEDVi had no statistically significant change between Time 1 and Time 2, but it significantly increased at Time 3 (*p* < 0.001). The LVEDVi values were as follows: Time 1: 47 ± 20; Time 2: 46 ± 16, and Time 3: 54 ± 25 mL/m^2^. For LVSVi there was a statistically significant increase from Time 1 to Time 3 (Table 3, Figure 1 (bottom)). The LVSVi was as follows: Time 1: 30 ± 26; Time 2: 27 ± 9; and 3: 34 ± 14 mL/m^2^. For the mitral valve E/E′, there was a statistically significant increase from Time 1 to Time 2 and a statistically significant decrease from Time 2 to Time 3 (*p* < 0.01). There was no significant change between Time 1 and Time 3. The mitral valve E/E′ values were as follows: Time 1: 7 ± 3; Time 2: 11 ± 6; and Time 3: 7 ± 2 (Table 3).

### 3.5. Effect of Previous Glenn Operation on Ventricular Function

Twenty-five patients had a Glenn operation before the Cone procedure. On the discharge echocardiogram (Time 2), patients with a prior Glenn had significantly higher LVEDVi (56.8 ± 18.9 vs. 42.5 ± 13.0 mL/m^2^, *p* < 0.001), higher LVSVi (32.9 ± 10.5 vs. 26.0 ± 8.0 mL/m^2^, *p* = 0.002), lower LVEF (58.3 ± 5.3 vs. 61.9 ± 5.5%, *p* = 0.004) compared to those without a Glenn. By Time 3, LVEDVi and LV function measures were no longer significantly different, but TAPSE was slightly lower in the Glenn group (1.0 ± 0.4 vs. 1.4 ± 0.4 cm, *p* = 0.044) (Table 4).

### 3.6. Effect of Previous Starnes Operation on Ventricular Function

Fourteen patients had the Starnes procedure prior to the Cone operation. Patients with the Starnes procedure had significantly lower RV FAC, TAPSE, and Tricuspid S′ before the operation at Time 1 (*p* < 0.001). At Time 2, the patients with the Starnes procedure had higher LVEF, LV stroke volume, LVEDVi, and RV FAC. TAPSE remained lower at Time 2. At Time 3, LVEF continued to be higher in patients with prior Starnes (Table 4).

### 3.7. Effect of Ventricular Dilation on Ventricular Function

Patients with moderate or severe RV dilation had significantly lower LVEDVi (41.0 ± 15.9 vs. 52.9 ± 13.5 mL/m^2^, *p* < 0.001), LVSVi (24.8 ± 9.4 vs. 32.2 ± 7.6 mL/m^2^, *p* < 0.001), and RV FAC (18.2 ± 8.2 vs. 26.9 ± 8.5%, *p* < 0.001) on discharge echocardiogram (Time 2) compared to those with mild or no dilation. However, by Time 3, there were no statistically significant differences in RV or LV functional parameters between the two groups (Table 5).

### 3.8. Analyzing Patients That Had Functional Parameters at All Three Time Points

**Figure 2:**
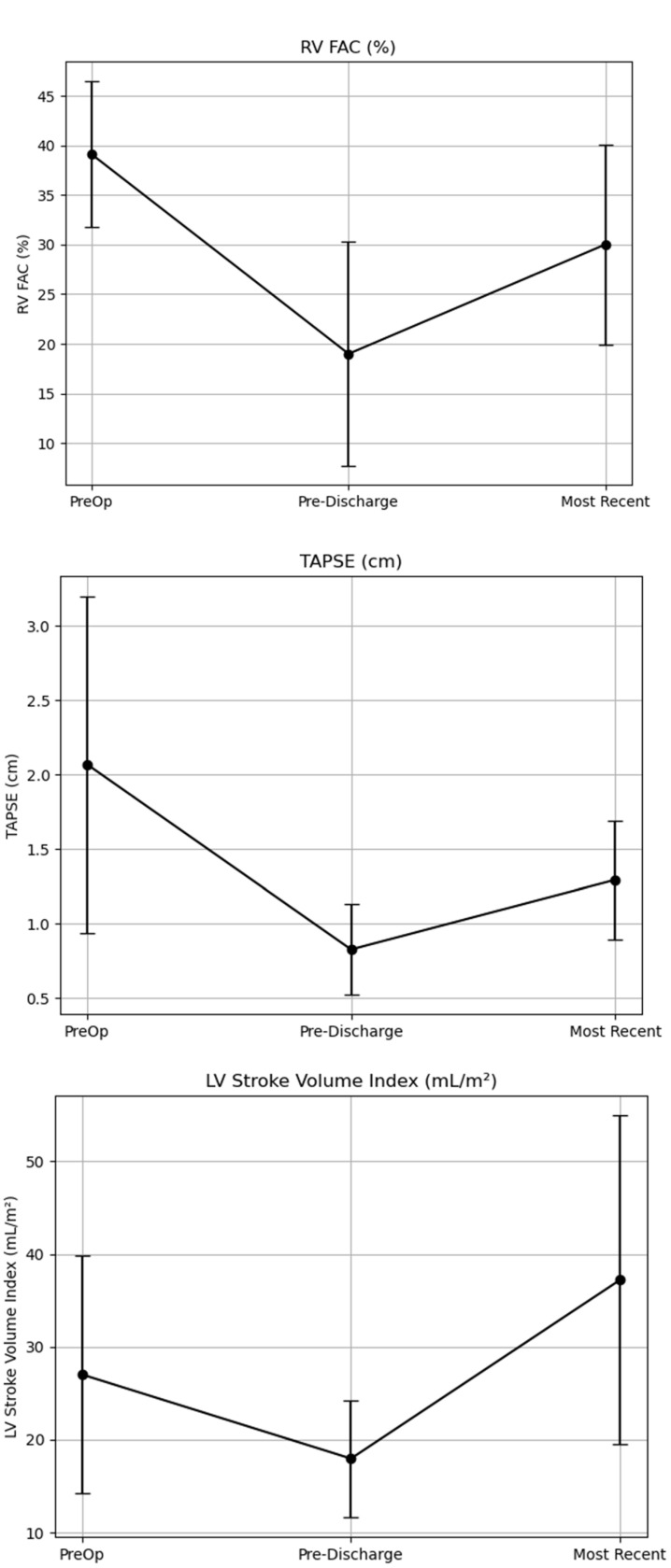
This figure shows the data for patients that had data recorded at all three time points with no missing data points. (**Top**) panel shows the RV FAC at the three different time points, with worsening at Time 2 and improvement at Time 3 (*p* < 0.05). (**Middle**) panel shows the TAPSE at the three different time points with worsening at Time 2 and improvement at Time 3 (*p* < 0.001). (**Bottom**) panel shows the LVSVi at the three different time points with improvement from Time 1 to Time 3 (*p* < 0.04).

Given that there were patients lost to follow up, a subgroup analysis was performed of patients that had data points available at all three time points. For the RV FAC there were nine patients available for all three time points. For TAPSE there were 37 patients available at all three time points. For the LVSVi there were 17 patients that had data at all three time points. RV FAC was decreased at Time 2 with partial recovery at Time 3 (*p* < 0.05). TAPSE declined at Time 2 and improved slightly by Time 3 (*p* < 0.001). LVSVi decreased at Time 2 but increased significantly at Time 3 compared to the pre-discharge period (*p* < 0.05). This is demonstrated in Figure 2.

## 4. Discussion

In this study of 134 patients who underwent the Da Silva Cone procedure for Ebstein anomaly, biventricular systolic function was systematically evaluated at three time points: preoperatively, at hospital discharge, and at least six months postoperatively. There was a significant and immediate decline in RV function postoperatively, with partial recovery at Time 3 during interval follow-up. Meanwhile, LVEDVi and LVSVi progressively increased by mid-term follow-up, but LVEF remained stable (graphical abstract). Subgroup analysis revealed that patients with prior Glenn or Starnes procedures had distinct early functional profiles, and patients with moderate or severe RV dilation showed worse early postoperative function, which later improved.

The acute postoperative deterioration in RV function following the Cone procedure is well-documented and was consistent across all RV metrics in our study—FAC, TAPSE, and tricuspid S′ [8,11]. This immediate decline is attributed to several factors. First, there are inherent structural abnormalities of the RV in Ebstein anomaly, particularly the thin, dysplastic, atrialized segment and the portion where the tricuspid valve failed to delaminate from the ventricular wall. Second, surgical incorporation of this atrialized segment into the functional RV can transiently worsen compliance and systolic function. Third, the postoperative reduction in TR often causes an acute increase in preload, which overwhelms the thin, often poorly contractile, right ventricle, further contributing to RV dysfunction. Lastly, cardiopulmonary bypass and myocardial manipulation during valve delamination may cause ischemia or conduction abnormalities, such as right bundle branch block, further compromising RV output [8,11,12,13].

This study reassuringly confirms that postoperative RV dysfunction after Cone procedure significantly improves over time. These findings are consistent with previous echocardiographic findings showing partial improvement of RV function over time [5]. By Time 3, all RV function parameters studied demonstrated improvement, though they did not fully return to preoperative values. This partial recovery likely reflects myocardial remodeling, adaptation of the restructured RV, and hemodynamic unloading from corrected TR. Prior to the Cone procedure, there is often massive RV volume overload secondary to TR, leading to shifting of the interventricular septum into the LV cavity with subsequent reduction in LV filling and function. In the immediate postoperative period, the RV remains dysfunctional since it is now pumping blood from a lower pressure (right atrium) to a higher pressure (pulmonary artery) structure. The interventricular septum slowly shifts back into place over time, and the RV remodels with improved contractility. This return of the interventricular septum to the midline in the long-term postoperative period improves both the RV and LV function, leading to improvement in function parameters [14]. Similarly, LV dysfunction post-mitral valve repair is a well-documented phenomenon due to alterations of preload and afterload post-operatively [15].

However, after Cone repair, the LV demonstrated favorable remodeling in our study with significant increases in LVEDVi and stroke volume, suggesting improved left heart filling due to favorable interventricular septal shift and improved RV stroke volume, enhancing LV preload and performance. Importantly, LVEF remained preserved, supporting that the procedure does not adversely affect LV systolic function. Previous CMR studies have shown similar findings with increased LVEDVi and LVSVi post-Cone operation [3,16].

We further examined how prior surgical interventions influenced outcomes. Patients with a history of Glenn shunt or Starnes palliation had significantly different baseline and early postoperative function. Those with Glenn had higher early LV volumes and lower TAPSE at follow-up but no other differences, suggesting the favorable impact of the Cone operation as part of the one and a half ventricle strategy. Meanwhile, patients with prior Starnes showed lower RV function preoperatively as expected and at discharge, but higher LVSVi and LVEF. Lower preoperative TAPSE, higher pre-discharge LVEF, and higher LV volumes were seen in patients with Glenn while higher LV volumes were seen in patients with preoperative Starnes. In patients with Glenn, this could be due to the reduced volume seen by the right heart with redirection of the superior vena caval blood flow directly to the pulmonary arteries. Similarly, patients with Starnes have reduced blood flow through the right heart due to exclusion of the right ventricle, allowing for improved blood flow through the left ventricle. This then would be similar at follow-up because the RV cavity becomes smaller, allowing for similar improvement in LV mechanics. Notably, by Time 3, most functional differences between surgical subgroups had resolved, reflecting the capacity of the remodeled heart to normalize function over time. The Cone after Starnes operation allows rehabilitation of the RV with excellent recovery of the RV function [5,16].

Lastly, significant RV dilation at baseline strongly influenced early postoperative ventricular function. Patients with moderate or severe RV dilation had significantly lower FAC and LVSVi at discharge. This reinforces the idea that preoperative RV geometry and load significantly affect postoperative recovery. However, at ≥6 months, these differences were no longer statistically significant, again underscoring the heart’s capacity for functional improvement following surgical correction [14].

### Limitations

The main limitation of this study was the number of patients that had echocardiograms at Time 3. Given that our institution is an international referral center for the Cone procedure, many patients travel to have the procedure performed, but follow up at their local hospitals after discharge from our institution, which resulted in missing follow-up data at Time 3. Another limitation was the inability to retrieve all data elements listed on each echocardiogram report as not all measurements could be performed due to anatomic abnormalities or post-surgical changes. Lastly, echocardiogram measurements of RV parameters are not as reliable as those on MRI. However, echocardiograms are more commonly and readily obtained than cardiac MRIs, which makes this study more applicable to clinical practice. Given the young age of many of the patients, utilizing CMRI would require multiple sedations to perform pre-, post-, and long-term follow-up studies. This was not practical given the study’s retrospective design.

## 5. Conclusions

The Da Silva Cone procedure is associated with an early decline in RV function due to the anatomical complexity and surgical demands of Ebstein anomaly. This study demonstrates mid-term improvement of RV systolic performance and a significant increase in LV filling and stroke volume compared to baseline. These changes likely reflect favorable ventricular remodeling and enhanced interventricular interaction following the relief of severe TR. Importantly, prior Glenn or Starnes procedures did not result in persistent RV dysfunction following the Cone. Patients with significant preoperative RV dilation exhibited worse RV function immediately postoperatively, but demonstrated recovery over time. These findings support the role of longitudinal echocardiographic assessment in monitoring recovery and guiding post-Cone management strategies.

## Figures and Tables

**Table 1:** Patient demographics.

Patient Characteristics	Median (Interquartile Range) or % (n)
Median Age of Surgery (years)	7.8 years (IQR): 2.3–17.7)
Previous Cardiac Surgery (%)	29% (n = 39)
Males (%)	47.8% (n = 64)
Females (%)	52.2% (n = 70)

Demographics: These demographics include mean age of surgeries, previous surgeries, abnormal EKG, and sex.

**Table 2:** Types of previous procedure or catheterization.

Previous surgery or Catheterization Procedure *	Percentage (n)
Starnes	10% (n = 14)
Blalock–Taussig–Thomas Shunt	17% (n = 23)
Central Shunt (Aorta to MPA)	5% (n = 7)
Patent Ductus Arteriosus Stent	4% (n = 6)
Glenn	19% (n = 25)
Previous Cone	4% (n = 6)
ASD Device Closure	6% (n = 8)
ASD Surgical Closure	6% (n = 8)
Ablation	23% (n = 31)
TV Repair	4% (n = 6)
TV Replacement	1% (n = 2)
PV Repair	2% (n = 3)
PV Replacement	1% (n = 2)
Other *	22% (n = 29)

This table illustrates the different types of surgeries/procedures that were performed prior to the Cone procedure. A total of 39 patients (29%) underwent an intervention before the Cone operation. (MPA, main pulmonary artery; ASD, atrial septal defect; TV, tricuspid valve; PV, pulmonary valve; VSD: ventricular septal defect; PDA, patent ductus arteriosus). * The other procedures included MPA ligation, VSD closure, PDA closure, and right atrial reduction.

**Table 3:** Echo function parameters at Times 1, 2, and 3.

Echo Parameter	Time 1 (Pre-Op)	Time 2 (Pre-Discharge)	Time 3 (Most Recent)	Time 1 vs. Time 2 (*p*-Value)	Time 1 vs. Time 3 (*p*-Value)	Time 2 vs. Time 3 (*p*-Value)
RV Fractional Area of Change (%)	35 ± 10 (n = 53)	21 ± 10 (n = 79)	29 ± 10 (n = 35)	<0.001	<0.001	0.039
TAPSE (cm)	2.0 ±1.0 (n = 118)	0.8 ± 0.3 (n = 118)	1.3 ± 0.4 (n = 54)	<0.001	<0.001	<0.001
Tricuspid S′ (cm/s)	13 ± 6 (n = 95)	5 ± 4 (n = 95)	7 ± 2 (n = 39)	<0.001	<0.001	0.115
LVEF (%)	59 ± 6 (n = 129)	61 ± 6 (n = 122)	61 ± 3 (n = 64)	0.011	0.227	0.988
LV EDVi (mL/m^2^)	47 ± 20 (n = 99)	46 ± 16 (n = 87)	54 ± 25 (n = 45)	0.408	0.033	0.033
LVSVi (mL/m^2^)	30 ± 26 (n = 98)	27 ± 9 (n = 98)	34 ± 14 (n = 39)	0.279	0.005	0.027
MV E/E′	7 ± 3 (n = 110)	11 ± 6 (n = 105)	7 ± 2 (n = 15)	<0.001	0.885	0.008

This table demonstrates the echo function parameters at Time 1 (preoperative), Time 2 (pre-discharge), and Time 3 (most recent). It then shows the *p*-values for each of the paired sample *t*-tests performed.

**Table 4:** Effect of Glenn and Starnes operation on ventricular function.

Time 1 (n = 129)	No Glenn (Mean ± SD) N = 106	Glenn (Mean ± SD) N = 23	*p*-Value	No Starnes (Mean ± SD) N = 115	Starnes (Mean ± SD) N = 14	*p*-Value
LVEDVi (mL/m^2^)	43.2 ± 15.6	65.1 ± 25.9	<0.001	43.4 ± 16	72.0 ± 22.0	<0.001
LVSVI (mL/m^2^)	28.5 ± 28.4	39.3 ± 16.9	0.123	28.5 ± 28.1	43.4 ± 11.8	0.063
LVEF (%)	59.3 ± 6.3	60.0 ± 6.1	0.642	59.3 ± 6.3	60.4 ± 6.0	0.535
RV FAC (%)	35.9 ± 9.8	26.9 ± 8.6	0.027	36.6 ± 8.3	22.5 ± 13.0	<0.001
TAPSE (cm)	2.4 ± 1.0	1.0 ± 0.4	0.001	2.335 ± 1.0	0.7 ± 0.2	<0.001
Tricuspid S′ (cm/s)	13.6 ± 5.5	7.3 ± 3.9	0.001	13.4 ± 5.5	4.8 ± 1.8	<0.001
**Time 2 (n = 122)**	**N = 106**	**N = 23**		**N = 115**	**N = 14**	
LVEDVi (mL/m^2^)	42.5 ± 13.0	56.8 ± 18.9	<0.001	43.7 ± 14.8	59.9 ± 14.7	0.001
LVSVI (mL/m^2^)	26.0 ± 8.0	32.9 ± 10.5	0.002	26.6 ± 9.0	34.2 ± 7.8	0.009
LVEF (%)	61.9 ± 5.5	58.3 ± 5.3	0.004	57.0 ± 5.1	61.7 ± 5.5	0.003
RV FAC (%)	21.2 ± 9.1	19.6 ± 11.7	0.555	20.0 ± 9.3	28.0 ± 9.7	0.018
TAPSE (cm)	0.79 ± 0.3	0.7 ± 0.2	0.105	0.8 ± 0.3	0.5 ± 0.2	0.001
Tricuspid S′ (cm/s)	5.22 ± 4.4	4.1 ± 1.4	0.237	5.1 ± 4.0	4.2 ± 1.5	0.439
**Time 3 (n = 64)**	**N = 55**	**N = 24**		**N = 59**	**N = 5**	
LVEDVi (mL/m^2^)	55.4 ± 24.5	43.4 ± 30.5	0.285	54.6 ± 24.6	47.2 ± 36.6	0.544
LVSVI (mL/m^2^)	34.6 ± 13.7	33.5 ± 14.7	0.865	33.9 ± 13.8	39.5 ± 12.7	<0.001
LVEF (%)	60.8 ± 6.1	61.2 ± 10.1	0.877	60.3 ± 6.5	67.6 ± 6.2	0.020
RV FAC (%)	29.9 ± 10.3	19.8 ± 6.3	0.183	N/A	N/A	N/A *
TAPSE (cm)	1.36 ± 0.4	1.0 ± 0.4	0.044	1.4 ± 0.4	0.8 ± 0.5	0.008
Tricuspid S′ (cm/s)	6.85 ± 1.8	7.1 ± 2.8	0.826	6.9 ± 1.9	5.7 ± 1.4	0.257

This table illustrates the effects of the Glenn and Starnes operation on ventricular function parameters at Time 1, Time 2, and Time 3. * There was only one patient with data for RV FAC Starnes at Time 3, so we were unable to perform the analysis.

**Table 5:** Right ventricular dilation and ventricular function parameters.

Time 1			
Parameter	Mild RV Dilation (N = 36)	Moderate–Severe RV Dilation (N = 67)	*p*-Value
LVEDVi (mL/m^2^)	45.6 ± 19.1	44.7 ± 15.3	0.823
LVSVi (mL/m^2^)	27.5 ± 11.1	31.2 ± 36.2	0.598
LVEF (%)	60.2 ± 7.5	58.9 ± 4.9	0.294
RV FAC (%)	35.0 ± 15.3	35.1 ± 6.8	0.976
TAPSE (cm)	1.7 ± 1.0	2.4 ± 1.0	0.009
S′ (cm/s)	10.5 ± 5.7	14.1 ± 5.3	0.009
**Time 2**			
**Parameter**	**Mild RV Dilation (N = 43)**	**Moderate–Severe RV Dilation (N = 64)**	***p*-Value**
LVEDVi (mL/m^2^)	52.9 ± 13.5	41.0 ± 15.9	<0.001
LVSVi (mL/m^2^)	32.2 ± 7.6	24.8 ± 9.4	<0.001
LVEF (%)	61.7 ± 5.0	61.5 ± 5.3	0.807
RV FAC (%)	26.9 ± 8.5	18.2 ± 8.2	<0.001
TAPSE (cm)	0.72 ± 0.3	0.81 ± 0.3	0.135
S′ (cm/s)	4.40= ± 1.53	5.46 ± 4.8	0.215
**Time 3**			
**Parameter**	**Mild RV Dilation (N = 32)**	**Moderate–Severe RV Dilation (N = 28)**	***p*-Value**
LVEDVi (mL/m^2^)	46.6 ± 22.6	59.3 ± 27.2	0.113
LVESVi (mL/m^2^)	20.3 ± 8.1	25.1 ± 10.4	0.129
LVSVi (mL/m^2^)	31.5 ± 10.1	36.9 ± 16.5	0.240
LVEF (%)	61.2 ± 5.5	60.1 ± 8.0	0.539
RV FAC (%)	31.2 ± 8.4	27.5 ± 12.8	0.325
TAPSE (cm)	1.37 ± 0.4	1.30 ± 0.34	0.536
S′ (cm/s)	6.55 ± 1.9	7.29 ± 1.8	0.242

This table shows differences in ventricular function parameters based on degree of right ventricular dilation.

## Data Availability

The data that supports the findings of this study are available from the corresponding author upon reasonable request.

## Abbreviations

The following abbreviations were used in this paper:

LVEF	Left Ventricular Ejection Fraction
RV	Right Ventricle
LV	Left Ventricle
TR	Tricuspid Regurgitation
UPMC	University of Pittsburgh Medical Center
MPA	Main Pulmonary Artery
ASD	Atrial Septal Defect
TV	Tricuspid Valve
PV	Pulmonary Valve
VSD	Ventricular Septal Defect
PDA	Patent Ductus Arteriosus
EF	Ejection Fraction
BSA	Body Surface Area
LVEDVi	BSA Indexed End-Diastolic Volume
Mitral valve	E/E′
LVSVi	BSA Indexed LV Stroke Volume
FAC	Fractional Area Change
TAPSE	Tricuspid Annular Plane Systolic Excursion
